# Nonlocal Intracranial Cavity Extraction

**DOI:** 10.1155/2014/820205

**Published:** 2014-09-28

**Authors:** José V. Manjón, Simon F. Eskildsen, Pierrick Coupé, José E. Romero, D. Louis Collins, Montserrat Robles

**Affiliations:** ^1^Instituto de Aplicaciones de las Tecnologías de la Información y de las Comunicaciones Avanzadas (ITACA), Universitat Politècnica de València, Camino de Vera s/n, 46022 Valencia, Spain; ^2^Center of Functionally Integrative Neuroscience, Department of Clinical Medicine, Aarhus University, Nørrebrogade 44, 8000 Aarhus, Denmark; ^3^Laboratoire Bordelais de Recherche en Informatique, Unité Mixte de Recherche CNRS (UMR 5800), PICTURA Research Group, 351 Cours de la Libération, 33405 Talence cedex, France; ^4^McConnell Brain Imaging Centre, Montreal Neurological Institute, McGill University, 3801 University Street, Montreal, QC, Canada

## Abstract

Automatic and accurate methods to estimate normalized regional brain volumes from MRI data are valuable tools which may help to obtain an objective diagnosis and followup of many neurological diseases. To estimate such regional brain volumes, the intracranial cavity volume (ICV) is often used for normalization. However, the high variability of brain shape and size due to normal intersubject variability, normal changes occurring over the lifespan, and abnormal changes due to disease makes the ICV estimation problem challenging. In this paper, we present a new approach to perform ICV extraction based on the use of a library of prelabeled brain images to capture the large variability of brain shapes. To this end, an improved nonlocal label fusion scheme based on BEaST technique is proposed to increase the accuracy of the ICV estimation. The proposed method is compared with recent *state-of-the-art* methods and the results demonstrate an improved performance both in terms of accuracy and reproducibility while maintaining a reduced computational burden.

## 1. Introduction

Automated brain image analysis has a huge potential to objectively help in the diagnosis and followup of many neurological diseases. To perform such analysis tasks, one of the first image processing operations is the delimitation of the area of interest. For brain image analysis, this operation has received many different names such as brain extraction, skull stripping, or intracranial cavity masking. In each case, the aim is to isolate the brain or intracranial tissues (depending on area definition) from the raw image. The accurate estimation of the intracranial volume plays crucial role to obtain robust and reliable normalized measurements of brain structures [1].

The importance of this operation is reflected by the large number of methods proposed over the past decade [2–13]. Many of these methods are based on the modeling of brain intensities (normally using T1 weighted images due to their excellent contrast for brain tissues) combined with a set of morphological operations [3, 5, 12] or atlas priors [9].

The most widely used automated methods correspond to those that are publically available. For example, the BET (brain extraction tool) software from the FSL image processing library [2] is one of the most used techniques probably due to its accuracy, ease of use, and low computational load. Other techniques like 3dIntracranial [6], hybrid watershed algorithm (HWA) [5], or brain surface extractor (BSE) [13] have been also widely used.

Intracranial cavity extraction can also be obtained indirectly as part of a full modeling of brain intensities using a parametric model such as that done in Statistical Parametric Mapping (SPM) [14] or VBM8 (http:/dbm.neuro.uni-jena.de/vbm/) software packages.

Over the last decade, methods have been proposed to automatically measure the intracranial cavity volume (ICV) by using nonlinear registration atlas-based approaches [15, 16].

More recent works of special interest for the brain extraction problem are methods like MAPS [10] and BEaST [11]. Both methods rely on the application of a multiatlas label fusion strategy. MAPS uses multiple nonlinear registrations followed by a voxel-wise label fusion while BEaST uses a single linear registration in combination with nonlocal patch-based label fusion. Both techniques scored well on the LONI segmentation validation engine (SVE) [17] comparison for brain extraction (see http://sve.bmap.ucla.edu/archive/) although MAPS has a much larger computational load compared to BEaST.

In this paper we present an extension of the BEaST methodology where we aim to improve the accuracy while reducing the computational load. The main contributions of the proposed method are threefold: first, the use of a new pipeline for the multiatlas library construction for improved normalization between template library subjects; second, the use of a new bilateral patch similarity measure to better estimate pattern similarities; and finally, a blockwise labeling approach that enables significant savings in computational cost and imposing at the same time a regularization constraint that increases the method's accuracy.

## 2. Materials and Methods

Since the method proposed in this paper is based on the use of a library of prelabeled cases to perform the segmentation process, we will first describe the template library construction and then present the proposed method.

### 2.1. Template Library Construction

#### 2.1.1. Library Dataset Description

A library of manually labeled templates was constructed using subjects from different publically available datasets. To include as large age range as possible, different datasets nearly covering the entire human lifespan were considered. MRI data from the following databases were used.

*(i) Normal Adults Dataset*. Thirty normal subjects (age range: 24–75 years) were randomly selected from the open access IXI dataset (http://www.brain-development.org/). This dataset contains images from nearly 600 healthy subjects from several hospitals in London (UK). Both 1.5 T (7 cases) and 3 T (23 cases) images were included in this dataset. 3 T images were acquired on a Philips Intera 3 T scanner (TR = 9.6 ms, TE = 4.6 ms, flip angle = 8°, slice thickness = 1.2 mm, volume size = 256 × 256 × 150, voxel dimensions = 0.94 × 0.94 × 1.2 mm^3^). 1.5 T images were acquired on a Philips Gyroscan 1.5 T scanner (TR = 9.8 ms, TE = 4.6 ms, flip angle = 8°, slice thickness = 1.2 mm, volume size = 256 × 256 × 150, voxel dimensions = 0.94 × 0.94 × 1.2 mm^3^).

*(ii) Alzheimer's Disease (AD) Dataset*. Nine patients with Alzheimer's disease (age range = 75–80 years, MMSE = 23.7 ± 3.5, CDR = 1.1 ± 0.4) scanned using a 1.5 T General Electric Signa HDx MRI scanner (General Electric, Milwaukee, WI) were randomly selected. This dataset consisted of high resolution T1-weighted sagittal 3D MP-RAGE images (TR = 8.6 ms, TE = 3.8 ms, TI = 1000 ms, flip angle = 8°, slice thickness = 1.2 mm, matrix size = 256 × 256, voxel dimensions = 0.938 × 0.938 × 1.2 mm^3^). These images were downloaded from the brain segmentation testing protocol [18] website (https://sites.google.com/site/brainseg/) while they belong originally to the open access OASIS dataset (http://www.oasis-brains.org/).

*(iii) Pediatric Dataset*. Ten infant datasets were also downloaded from the brain segmentation testing protocol [18] website (https://sites.google.com/site/brainseg/). These data were originally collected by Gousias et al. [19] and are also available at http://www.brain-development.org/ (this dataset is property of the Imperial College of Science Technology & Medicine and has been used after accepting the license agreement). The selected 10 cases are from the full sample of 32 two-year-old infants born prematurely (age = 24.8 ± 2.4 months). Sagittal T1 weighted volumes were acquired from each subject (1.0 T Phillips HPQ scanner, TR = 23 ms, TE = 6 ms, slice thickness = 1.6 mm, matrix size = 256 × 256, voxel dimensions = 1.04 × 1.04 × 1.6 mm^3^ resliced to isotropic 1.04 mm^3^).

Downloaded images from the different websites consisted of raw images with no preprocessing and no intracranial cavity masks were supplied with these data. To generate the template library, all 49 selected T1-weighted images were preprocessed as follows.

#### 2.1.2. Denoising and Inhomogeneity Correction

All images in the database were denoised using the spatially adaptive nonlocal means (SANLM) filter [20] to enhance the image quality. The SANLM filter can deal with spatially varying noise levels across the image without the need of explicitly estimating the local noise level which makes it ideal to process data with either stationary or spatially varying noise (as in the case of parallel imaging) in a fully automatic manner. To further improve the image quality, an inhomogeneity correction step was applied using the N4 method [21]. The N4 method is an incremental improvement of the N3 method [22] that has been implemented in the ITK toolbox [23] and has proven to be more efficient and robust.

#### 2.1.3. MNI Space Registration

In order to perform the segmentation process, templates and the subject to be segmented have to be placed in the same stereotactic space. Therefore, a spatial normalization based on a linear registration to the Montreal Neurological Institute (MNI 152) space was performed using ANTS software [24]. The resulting images in the MNI space have a size of 181 × 217 × 181 voxels with 1 mm^3^ voxel resolution.

#### 2.1.4. Intensity Normalization

**Figure 1:**
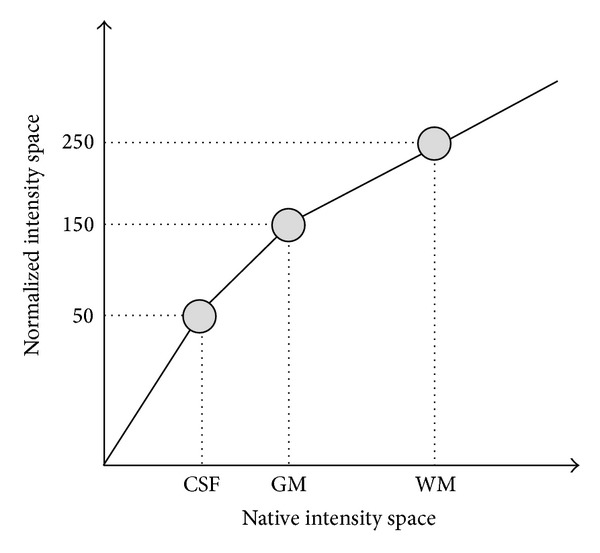
Proposed intensity normalization via a piecewise linear mapping. CSF, GM, and WM mean values are automatically estimated using TMS method and mapped to their corresponding normalized values (50, 150, and 250).

As the proposed method is based on the estimation of image similarities using intensity-derived measures, every image in the library must be intensity normalized in order to make the intensity distributions comparable among them. We use a tissue-derived approach to force mean intensities of white matter (WM), grey matter (GM), and cerebrospinal fluid (CSF) to be as similar as possible across subjects of the library in a similar manner as done by Lötjönen et al. [25]. For this purpose, mean values of CSF, GM, and WM tissues were estimated using the trimmed mean segmentation (TMS) method [26] which robustly estimates the mean values of the different tissues by excluding partial volume voxels from the estimation jointly with the use of an unbiased robust mean estimator. Such estimation was performed using only voxels within the standard brain mask area of MNI 152 template to minimize the inclusion of external tissues. Finally, a piecewise linear intensity mapping [25, 27] was applied ensuring that WM had an average intensity of 250, GM of 150, and CSF of 50 (see Figure 1).

#### 2.1.5. Manual Labeling

As commented previously, there is no standard definition of what should be included in brain or intracranial masks (it all depends on what you are looking for). In BEaST, the mask definition included the following tissues:

- all cerebral and cerebellar white matters,
- all cerebral and cerebellar gray matters,
- CSF in ventricles (lateral, 3rd, and 4th) and the cerebellar cistern,
- CSF in deep sulci and along the surface of the brain and brain stem,
- the brainstem (pons, medulla),
- internal brain blood vessels.

In the present work we extended that definition by including all external CSF (thus covering total CSF of IC) and therefore selecting most of the intracranial cavity volume. We have not included other intracranial tissues in our mask definition such as dura, exterior blood vessels, or veins because they are normally of no interest for brain analysis. This mask definition has been traditionally used to estimate the total intracranial volume (TIV) in many methods such RBM [28], SPM8, or VBM8 methods to normalize brain tissue volumes [29, 30] as it is expected to be nearly constant in each subject during the adult lifespan.

**Figure 2:**
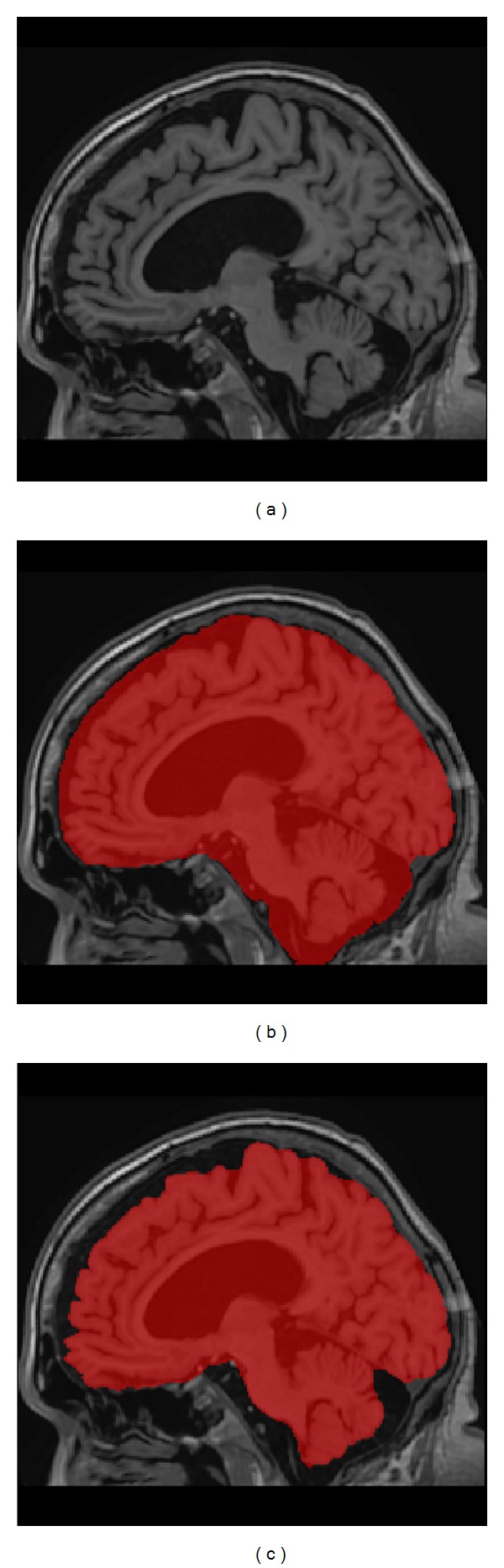
Example of mask differences between our mask definition (b) and BEaST mask (c) for an Alzheimer's case (a). As can be noticed, all external CSF is included in NICE mask while this is not case at the corresponding BEaST mask (example case from Oasis dataset).

To generate the template masks we followed a similar approach as described in BEaST paper since full manual labeling was too time consuming and error prone as discussed in Eskildsen et al. [11]. All template images in the library were automatically segmented using BEaST software to have an initial mask. Conditional mask dilation (only over CSF voxels) was applied to include external CSF not already included in the BEaST mask. Finally, all the images were manually corrected by an expert on brain anatomy using the ITK-SNAP software [31] to remove segmentation errors. In Figure 2 we show an example of our mask definition compared to BEaST definition for a patient with Alzheimer's disease.

To further increase the number of available priors on the library, all the cases were flipped along the midsagittal plane using the symmetric properties of the human brain yielding a total number of 98 labeled templates (original and flipped) as done in BEaST paper [11].

**Figure 3:**
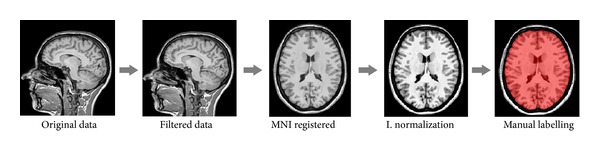
Template library construction pipeline.

Compared to BEaST template library creation, the main differences are the use of a denoising method to improve data quality, the use of a different registration method (ANTS instead of ANIMAL), and the application of different intensity normalization method. The scheme of the template library construction pipeline is summarized in Figure 3.

### 2.2. Proposed Method

While the BEaST technique was designed to improve downstream analysis such as the assessment of cortical thickness, our proposed method has extended the mask definition to include extracerebral spinal fluid as it can be interesting to obtain normalized brain and tissue specific volumes in many neurological diseases such as Alzheimer or Parkinson. We will refer our proposed method as NICE (nonlocal intracranial cavity extraction). Since the method proposed in this paper is an evolution of the BEaST brain masking method [11], we refer the reader to the original paper for the detailed method overview. Here, we summarize the NICE method and present the main improvements introduced to increase the method performance.

#### 2.2.1. Preprocessing

To segment a new case, it must be first preprocessed using the proposed normalization pipeline (see Section 2.1 and Figure 2) so that the new case is spatially aligned with the template library and to ensure that it has the same intensity characteristics.

#### 2.2.2. Improved Nonlocal Means Label Fusion

In the classical nonlocal means label fusion technique proposed by Coupé et al. [32], for each voxel *x*_*i*_ from the new image to be segmented the method estimates the final label by performing a weighted label fusion *v*(*x*_*i*_) of all surrounding samples inside the search area *V*_*i*_ from *N* subjects selected from the library:

(1) $$\begin{array}{c} v\left(x_{i}\right)=\frac{\sum_{s=1}^{N}\sum_{j\in V_{i}}w\left(x_{i},x_{s,j}\right)l_{s,j}}{\sum_{s=1}^{N}\sum_{j\in V_{i}}w\left(x_{i},x_{s,j}\right)}, \end{array}$$

where *l*_*s*,*j*_ is the label from the voxel *x*_*sj*_ at the position *j* in the template library subject *s* and *w*(*x*_*i*_, *x*_*s*,*j*_) is the weight calculated by patch comparison which is computed depending on the similarity of the surrounding patch for *x*_*i*_ and for *x*_*s*,*j*_ This weight is estimated as follows:

(2) $$\begin{array}{c} w\left(x_{i},x_{s,j}\right)=\{\begin{array}{cc} \exp^{{-||P(x_{i})-P(x_{s,j})||}_{2}^{2}/h^{2}}, & \text{if}\;\text{ss}<\text{th}\\ 0, & \text{otherwise}, \end{array} \end{array}$$

where *P*(*x*_*i*_) is the patch around the voxel *x*_*i*_, *P*(*x*_*s*,*j*_) is the patch around the voxel *x*_*j*_ in the templates, and ||·||_2_ is the normalized L2-norm (normalized by the number of elements) calculated by the distance between each pair of voxels from both patches *P*(*x*_*i*_) and *P*(*x*_*s*,*j*_) and modulated by *h* parameter. If ss (structural similarity index [33] between patches) is less than a threshold th, then *w* is not computed thus avoiding unneeded computations.

The structural similarity index ss is calculated as follows:

(3) $$\begin{array}{c} \text{ss}=\frac{2\mu_{i}\mu_{s,j}}{\mu_{i}^{2}+\mu_{s,j}^{2}}\cdot \frac{2\sigma_{i}\sigma_{s,j}}{\sigma_{i}^{2}+\sigma_{s,j}^{2}}, \end{array}$$

where *µ* and *σ* are the mean and standard deviation of the patches surrounding *x*_*i*_ and *x*_*s*,*j*_ at location *j* of the template *s*.

Finally, the final label *L*(*x*_*i*_) is computed as

(4) $$\begin{array}{c} L\left(x_{i}\right)=\{\begin{array}{cc} 1 & v\left(x_{i}\right)\geq 0.5\\ 0 & v\left(x_{i}\right)<0.5. \end{array} \end{array}$$

In this paper, we introduce two modifications to this strategy. First, we make use of the fact that all the images are registered to a common space and therefore a* locality principle* can be used, assuming that samples that are spatially closer are likely to be more similar in their labels. However, this locality principle is limited by residual anatomical variability and registration errors in the template library space. Therefore, we redefined the similarity weight to take into account not only intensity similarity but also spatial patch proximity:

(5) $$\begin{array}{c} w\left(x_{i},x_{s,j}\right)=\{\begin{array}{cc} \exp^{-[\left({||x_{i}-x_{j}||}_{}^{2}/\sigma_{d})+({||P(x_{i})-P(x_{s,j})||}_{2}^{2}/h\right)]}, & \\ \;\text{if}\;\text{ss}<\text{th} & \\ 0,\;\text{otherwise}, & \end{array} \end{array}$$

where *x*_*i*_ and *x*_*j*_ are the coordinates of patch centers and *σ*_*d*_ is normalization constant. We set *σ*_*d*_ = 8 mm experimentally which curiously coincides with the typical Gaussian blurring kernel size normally used on voxel based morphometry (VBM) to deal with registration error and subject anatomical variability. This approach shares some similarities to the bilateral filter proposed by Tomasi and Manduchi [34] for image denoising. We experimentally set the threshold th to 0.97 instead of 0.95 as used in BEaST (this difference can be explained due to the use of filtered data and a different intensity normalization method). Also a comment about *h* parameter of (5) is required since it plays a major role in the weight computation process. In [32] this value was set to

(6) $$\begin{array}{c} h\left(x_{i}\right)=\lambda \arg\underset{x_{s,l}}{\min}{||P\left(x_{i}\right)-P(x_{j,s})||}_{2}+\varepsilon ,\;\;\;\; \end{array}$$

where *ε* is a small constant to ensure numerical stability. In [32] *λ* was set to 1 but we found experimentally that a value of 0.1 produced better results in the proposed method possibly due to the improved intensity normalization.

The second modification concerns the voting scheme. Classical nonlocal label fusion works in a voxelwise manner which sometimes results in a lack of regularization on the final labels. Given that we wish to segment a continuous anatomical structure, some level of regularization can be used as a constraint achieved by a blockwise vote scheme, similar to the one used by Rousseau et al. [35] for label fusion and derived from MRI denoising [36]. This bilateral blockwise vote is computed as follows:

(7) $$\begin{array}{c} v\left(B\left(x_{i}\right)\right)=\frac{\sum_{s=1}^{N}\sum_{j\in V_{i}}w\left(x_{i},x_{s,j}\right)l\left(B\left(y_{s,j}\right)\right)}{\sum_{k=0}^{M}\sum_{s=1}^{N}\sum_{j\in V_{i}}w\left(x_{i},x_{s,j}\right)}, \end{array}$$

where *B*(*x*_*i*_) is a 3D region which is labeled at the same time. Finally, the vote for the voxel *x*_*i*_ is obtained in an overcomplete manner by averaging over all blocks containing *x*_*i*_ and the label *L*(*x*_*i*_) is decided as in (4).

With overlapping blocks, it is worth noting that the distance between adjacent block centers can be increased to be equal or higher than 2 voxels. Therefore, we can obtain important accelerating factors compared to the voxelwise version of the algorithm (e.g., for a distance equal to 2 voxels in all three directions a speedup factor of 2^3^ = 8 can be obtained). The described approach is used within the multiresolution framework as described in the BEaST paper [11].

## 3. Experiments

### 3.1. Experimental Datasets

To validate the proposed method, different datasets were used. These datasets can be classified two categories: (a) those that were used to measure the accuracy of the different methods compared and (b) those used to measure their reproducibility.

#### 3.1.1. Accuracy Datasets

*LOO Dataset*. To measure the accuracy of the proposed method, we used the template library dataset by using a leave-one-out (LOO) cross validation. The characteristics of this dataset have already been described in Section 2.1. Each of the 49 (nonflipped) library images was processed with the remaining images as priors (after removing the current case and its flipped version). The resulting segmentation was compared to the corresponding manual labels in the library.

*Independent Validation Dataset*. To avoid any factor associated to our IC mask definition that could bias the comparison of the compared methods, we decided to use an independent dataset with its corresponding manual segmentations. Therefore, we performed a validation using an independent dataset available in the online Segmentation Validation Engine (SVE) [17]. The SVE IC segmentation followed rules similar to those used here. This dataset consists of 40 T1w MRI scans and its associated manual labels (20 males and 20 females; age range 19–40). This high-resolution 3D Spoiled Gradient Echo (SPGR) MRI volume was acquired on a GE 1.5 T system as 124 contiguous 1.5 mm coronal brain slices (TR range 10.0 ms–12.5 ms; TE range 4.22 ms–4.5 ms; FOV 220 mm or 200 mm; flip angle 20°) with in-plane voxel resolution of 0.86 mm (38 subjects) or 0.78 mm (2 subjects).

#### 3.1.2. Reproducibility Dataset

Although the accuracy of a method is very important, another important feature is its reproducibility. Indeed, the capability to detect changes induced by the pathology in a consistent manner is a key aspect. To measure the reproducibility of the different compared methods, we used the reproducibility dataset of the brain segmentation testing protocol website (https://sites.google.com/site/brainseg/). This dataset consists of a test-retest set of 20 subjects scanned twice in the same scanner and sequence (SSS) and another set of 36 subjects scanned twice on different scanner and different magnetic field strength (DSDF) (1.5 and 3 Tesla).

*SSS Dataset*. To measure the reproducibility of the different methods compared on the same subjects and using the same MRI scanner, we used a subset of the OASIS (www.oasis-brains.org) dataset consisting in 20 subjects (age = 23.4 ± 4.0 years, 8 females) who were scanned using the same pulse sequence two times (1.5 T Siemens Vision scanner, TR = 9.7 ms, TE = 4 ms, TI = 20 ms, flip angle = 10°, slice thickness = 1.25 mm, matrix size = 256 × 256, voxel dimensions = 1 × 1 × 1.25 mm^3^ resliced to 1 × 1 × 1 mm^3^, averages = 1) [37].

*DSDF Dataset*. To determine the consistency of the segmentations when different MRI scanners and different magnetic field strength were used, 36 adult subjects were scanned using two MRI scanners (1.5 T and 3.0 T General Electric Signa HDx scanner), mean interscan interval between 1.5 T and 3 T scanner = 6.7 ± 4.2 days) [18].

### 3.2. Method Parameter Settings

**Figure 4:**
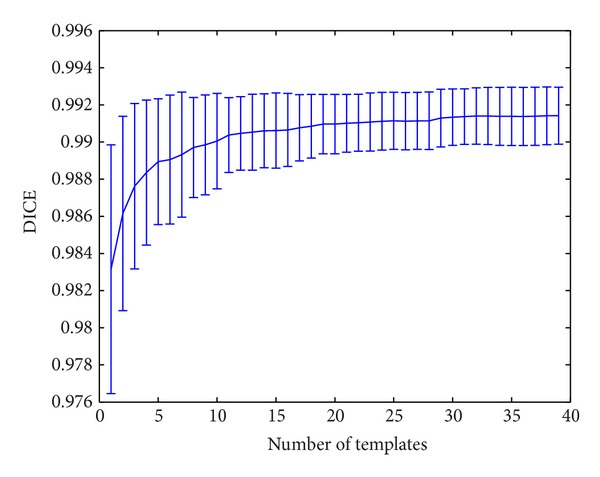
Evolution of segmentation accuracy in function of the number of training subject templates used in the segmentation process.

To study the impact of the method parameters, an exhaustive search of the optimum values was performed using the LOO dataset using the library segmentations as gold standard references. Each one of the 49 subjects in the library was processed using the remaining cases of the library as priors and the resulting segmentation was compared to the manual labeling. To measure segmentation accuracy, the Dice coefficient [38] was used. Method parameters such as patch size and search area were set as in BEaST method while an exhaustive search for the optimal number of templates *N* used for the segmentation process was carried out (see Figure 4). This search demonstrated that the segmentation accuracy stabilizes around *N* = [20–30] range which is in good agreement with previous results from BEaST. We decided to use *N* = 30 as default value given the reduced computational cost of the proposed method.

Another parameter of our proposed block-based approach is the spacing between adjacent blocks which jointly with patch size defines the degree of overlap between blocks. We observed experimentally that the optimal value for that parameter was 2 voxels in all 3 dimensions since the resulting accuracy was virtually the same from full overlap (1 voxel spacing) while computation time was greatly reduced. This is in good agreement with previous results on blockwise MRI denoising [36]. Higher block spacing resulted in worse segmentation results.

### 3.3. Compared Methods

The proposed method was compared with BEaST and VBM8 methods. Both BEaST and VBM8 methods were selected because of their public accessibility and because they are among the highest ranking methods on the online Segmentation Validation Engine website [17] (http://sve.bmap.ucla.edu/archivel/).

To ensure a fair comparison all three methods, we used the same preprocessing pipeline with the exception of the intensity normalization step (i.e., using ANTS registration to ensure the same image space and the same homogenization and filtering to ensure the same image quality). In this way, only the labeling process was evaluated eliminating other sources of variability.

Both NICE and BEaST were run with the same number of preselected templates (*N* = 30) to ensure a fair comparison. We used release 435 of VBM8, which was the latest version at the time of writing. To compare the segmentation results of the different methods, several quantitative metrics were used: DICE coefficient [38], sensitivity, and specificity.

## 4. Results

### 4.1. Accuracy Results

In Table 1, the average DICE coefficient, sensitivity, and specificity for all 49 cases of LOO dataset using the different methods compared are provided. Results for all the cases together and separated by dataset subtype are provided (Alzheimer's disease (AD), normal infants (infant), and normal adult subjects (adult)). As can be noticed, NICE method obtained the best results in all the situations.  Table 2 shows the statistical significance of these differences (paired *t*-test).

**Figure 5:**
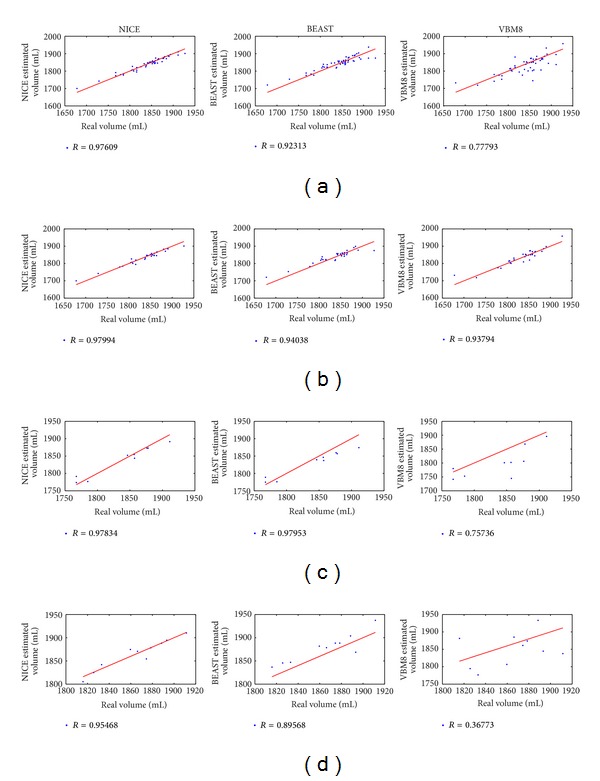
Comparison of intracranial cavity volume estimation results. Automatic versus manual volume correlation for all the compared methods and datasets used. The first row shows results for the whole library (*N* = 49), the second only for normal adults (*N* = 30), the third only for AD subjects (*N* = 9), and the fourth only for infant cases (*N* = 10). Red line represents ideal mapping between estimated and real volumes to highlight eventual over or under volume estimations (it does not represent the fitting line).

**Figure 6:**
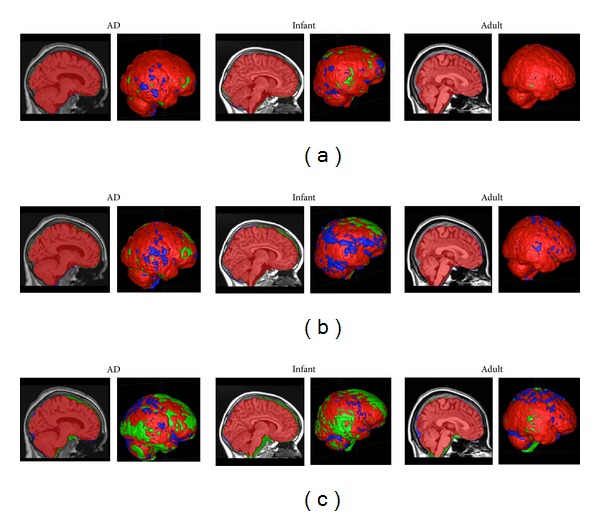
Example segmentation results using NICE (a), BEaST (b), and VBM8 (c) methods on the three different population samples. Sagittal slices and 3D renderings of the segmentations are shown. Red voxels correspond to correct voxels in the segmentation compared to the gold standard. Blue voxels are false positives and green voxels are false negatives (AD case belongs to Oasis dataset and the adult and infant cases belong to the IXI dataset).

Intracranial cavity volume is normally used to normalize brain tissue volumes to provide a tissue measure independent of head size. Therefore, the ability of the compared methods to provide an accurate ICV estimation has to be assessed. To this end, volume estimations using the different compared methods were obtained and compared to gold standard manual volumes.  Figure 5 shows the automatic versus manual volume correlation for all the compared methods and dataset used. As can be noticed, the NICE method had highest overall correlation (0.976) while BEaST and VBM8 had 0.923 and 0.778, respectively. In Figure 6, a visual comparison of the segmentation results of three examples belonging to the three different subject populations can be performed.

To perform an independent validation of the compared methods, the SVE dataset was used. The SVE web service allows the comparison of results with hand-corrected brain masks. As done in BEaST and MAPS papers, we used the brain masks provided by the SVE website which included all the internal ventricular CSF and some external sulcal CSF. Although this mask definition slightly differs from our mask definition (not all CSF was included), this does not represent a problem for the method's comparison since all the methods shared the same references.

**Figure 7:**
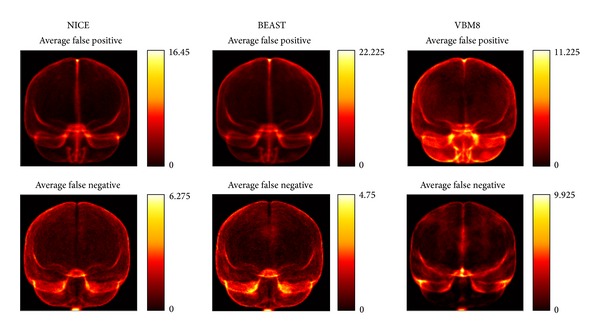
False positive and false negative maps for NICE, BEaST, and VBM8 on SVE dataset. VBM8 tended to produce a systematic oversegmentation compared to the used manual gold standard. The errors obtained by NICE and BEaST were more uniformly distributed indicating nonsystematic segmentation errors. Note that in the images provided by the SVE website the vertical scale measuring error is not the same over all images.

Validation of NICE using the SVE test dataset resulted in a mean DICE of 0.9819 ± 0.0024 (see http://sve.bmap.ucla.edu/archivel/). At the time of writing, this result was the best (*P* < 0.01) of all the methods published on the website. BEaST had a DSC of 0.9781 ± 0.0047 and VBM8 obtained a DSC of 0.9760 ± 0.0025. Sensitivity and specificity results are also included in Table 3. A visual representation of false positive and false negative as supplied by the website is presented at Figure 7.

### 4.2. Reproducibility Results

In Table 4, the average ICV differences for the different methods and datasets is provided. As can be noticed, NICE method obtained the most reproducible results in all situations. For the SSS dataset experiment (test-retest), NICE significantly improved BEaST method while these differences were not significant for VBM8 method. For the DSDF dataset experiment, volume differences were higher than in the previous experiment. In this case, NICE was found to yield significantly improved estimates (*P* < 0.05) compared to the two other methods.

Finally, execution times of the different methods were compared. NICE method took around 4 minutes (NICE was implemented as a multithreaded MEX C file), BEaST method took around 25 minutes (we have to note that no multithreading optimizations were used here), and VBM8 took around 8 minutes on average (in this time it was also included the different tissue segmentations). All the experiments were performed using MATLAB 2009b 64 bits (Mathworks, Inc.) on a desktop PC with an Intel Core i7 with 16 GB RAM running windows 7.

However, it is worth noting that if we reduce the number of selected templates to 10 cases, we can reduce the processing time to less than 1 minute with only a small reduction of the segmentation accuracy (0.9911 to 0.9901 in the LOO accuracy experiment).

## 5. Discussion

We have presented a new method for intracranial cavity extraction that outperforms related* state-of-the-art* methods and a previously proposed method (BEaST) by our group both in terms of accuracy and reduced computational load. In addition, we demonstrated that the new proposed method is more robust in terms of measurement reproducibility.

This last point is of special interest since in many cases we are not only interested in the specific brain volume at one time point but in its evolution in a longitudinal study. NICE method was demonstrated to be significantly more reproducible and accurate than BEaST method. In addition, VBM8 was found to be almost as reproducible as NICE but at the expense of introducing larger systematic errors on the segmentations. The high level of reproducibility of VBM8 may be explained by the fact that it uses a single template and thus a more deterministic pipeline is applied. Also the fact that it operates at 1.5 mm^3^ resolution introduces a blurring effect which increases the method reproducibility at the expense of the accuracy. The increased reproducibility/accuracy of our proposed method may have a significant impact on the brain image analysis methods by increasing their sensitivity to detect subtle changes produced by the disease. While the advantage of NICE in segmentation accuracy of 0.9911 versus 0.9762 for VBM8 may appear small when compared over the three datasets evaluated, it is statistically significant and corresponds to more than a 2-fold reduction in error, from 2.38% to 0.89%. In a large volume such as the intracranial cavity (1500 cc), this reduction in error can represent a volume of approximately 20 cc, a nonnegligible amount. The improvement over BEaST is smaller (35%) but still statistically significant. When evaluated on the SVE dataset, the NICE yields a Dice overall of 0.9819, while BEaST and VBM8 yield 0.9781 and 0.9760, corresponding to 20% and 32% less error on average, respectively.

The improved results of NICE over BEaST can be understood thanks to improvements on two parts of the proposed method. First, improvements on template library construction such as the improved intensity normalization yield more coherent and better defined priors. This fact positively impacts the intensity driven image similarities of the label fusion part. One limitation of the first part of our validation is in the use of manually corrected masks that may induce a favourable bias toward BEaST and NICE. However, after the conditional dilation and manual correction steps, almost all edge voxels were modified, thus minimizing any bias. Second, the blockwise and new bilateral label fusion scheme results in more regular and accurate segmentations. The advantages of using a 3D blockwise approach in comparison to the previously used voxelwise are twofold: first, the fact that we label together the whole block imposes an intrinsic regularization which forces connected voxels to have similar labels and second if a space between block centers is used, a significant speed-up factor can be obtained in comparison with the voxelwise version. Finally, the new similarity measure using spatial distance weighting takes into account a locality principle that favors the contribution of closer patches by assuming that after linear registration similar structures are close in a similar manner as done for the well-known bilateral filter for image denoising [34].

It is also worth noting that the segmentation accuracy depends on the preexistence of similar local patterns within the library. In our method, we do not need to have totally similar templates to the case to be segmented within the library since it is able to find locally similar patterns from different templates in the library. However, it is also true that if some specific pattern is not present in the library, it will not be correctly identified and therefore the resulting segmentation will be incorrect. This risk is normally reduced when using nonlinear registrations at the expense of a much higher computational load and the introduction of interpolation artifacts in both images and associated labels. However, this issue can be solved more efficiently (mainly in terms of computational cost) by increasing library size with uncommon cases and their associated corrected manual labels making it unnecessary to perform costly nonlinear registrations (but making necessary the manual label correction of new library cases). We experimentally found that increasing the size of the library just using the symmetric versions of the original library improved the segmentation results as previously reported in the BEAST paper. Finally, it is also possible to construct disease specific libraries (as done for templates in SPM) maximizing the likelihood to find suitable matches for the segmentation process or to improve templates preselection by adding extra information such as age or sex which could help to find optimal matches (especially useful when the library size will grow).

## 6. Conclusion

We have presented an improved method to perform intracranial cavity extraction that has been shown to be fast, robust, and accurate. The improvements proposed have been shown to increase segmentation quality and reduce the computational load at the same time (the proposed method is able to work in a reasonable time of approximately 4 minutes). We plan to make the NICE pipeline publicly accessible through a web interface in the near future so everybody can benefit from its use independently of their location and local computational resources. Finally, the usefulness of the proposed approach to provide accurate ICV based normalization brain tissue measurements has to be addressed on future clinical studies.

## Figures and Tables

**Table 1:** Average DICE coefficient for the different methods compared on the different used datasets. The best results from each column are in bold.

Method	Data	All (*N* = 49)	Adults (*N* = 30)	AD (*N* = 9)	Infants (*N* = 10)
NICE	DICE	**0.9911 ± 0.0020**	**0.9921 ± 0.0015**	**0.9892 ± 0.0016**	**0.9899 ± 0.0019**
	SEN	**0.9907 ± 0.0036**	**0.9916 ± 0.0035**	**0.9887 ± 0.0029**	0.9898 ± 0.0038
	SPE	**0.9971 ± 0.0012**	**0.9975 ± 0.0010**	**0.9964 ± 0.0015**	**0.9965 ± 0.0009**

BEAST	DICE	0.9880 ± 0.0032	0.9891 ± 0.0030	0.9857 ± 0.0018	0.9866 ± 0.0034
	SEN	0.9889 ± 0.0062	0.9902 ± 0.0060	0.9830 ± 0.0049	**0.9900 ± 0.0050**
	SPE	0.9955 ± 0.0019	0.9958 ± 0.0017	0.9960 ± 0.0016	0.9940 ± 0.0019

VBM8	DICE	0.9762 ± 0.0052	0.9788 ± 0.0026	0.9690 ± 0.0064	0.9750 ± 0.0033
	SEN	0.9740 ± 0.0121	0.9796 ± 0.0051	0.9587 ± 0.0132	0.9710 ± 0.0138
	SPE	0.9926 ± 0.0027	0.9924 ± 0.0019	0.9931 ± 0.0033	0.9926 ± 0.0041

**Table 2:** NICE compared to the other two methods (*P* values). Significant differences (*P* < 0.05) are in bold.

Method	Data	All (*N* = 49)	Adults (*N* = 30)	AD (*N* = 9)	Infants (*N* = 10)
BEAST	DICE	6.30 × 10^−8^	5.50 × 10^−6^	5.16 × 10^−4^	**0.014**
	SEN	0.074	0.283	**0.012**	0.913
	SPE	2.54 × 10^−6^	2.97 × 10^−5^	0.498	**0.002**

VBM8	DICE	1.26 × 10^−33^	3.97 × 10^−32^	3.35 × 10^−7^	3.29 × 10^−10^
	SEN	4.99 × 10^−15^	2.60 × 10^−15^	1.61 × 10^−5^	6.18 × 10^−4^
	SPE	2.06 × 10^−18^	2.73 × 10^−19^	**0.010**	**0.009**

**Table 3:** Segmentation results of SVE dataset using different quality measures. NICE was compared to the other two methods (*P* values). Best results are in bold (note that in this dataset, BEaST method obtained a significant higher sensitivity than NICE at the expense of a lower specificity).

Method	DICE	Sensitivity	Specificity
NICE	**0.9819 ± 0.0024**	0.9857 ± 0.0044	**0.9960 ± 0.0015**

BEAST	0.9781 ± 0.0047	**0.9887 ± 0.0035**	0.9940 ± 0.0025
	(*P* = 9.82 × 10^−7^)	(*P* = 0.0001)	(*P* = 2.11 × 10^−5^)

VBM8	0.9760 ± 0.0025	0.9840 ± 0.0046	0.9942 ± 0.0014
	(*P* = 2.56 × 10^−15^)	(*P* = 0.06)	(*P* = 4.31 × 10^−8^)

**Table 4:** Percent mean IC volume differences for the SSS dataset. NICE was compared to the other two methods (*P* values). Best results are in bold.

Method	SSS dataset	DSDF dataset
NICE	**1.4046 ± 0.2447**	**3.1856 ± 1.0280**

BEAST	2.1463 ± 0.6622	5.4696 ± 1.9097
	(*P* = 3.21 × 10^−4^)	(*P* = 1.55 × 10^−10^)

VBM8	1.4268 ± 0.3843	3.7741 ± 1.1627
	(*P* = 0.8073)	(*P* = 0.0242)
